# Hypotensive episodes at 24-h Ambulatory Blood Pressure Monitoring predict adverse outcomes in Parkinson’s Disease

**DOI:** 10.21203/rs.3.rs-3904996/v1

**Published:** 2024-02-06

**Authors:** Fabrizio Vallelonga, Matteo Valente, Marta Maria Tangari, Anna Covolo, Valeria Milazzo, Cristina Di Stefano, Gabriele Sobrero, Marta Giudici, Alberto Milan, Franco Veglio, Leonardo Lopiano, Simona Maule, Alberto Romagnolo

**Affiliations:** Universita degli Studi di Torino Dipartimento di Scienze Mediche; Universita degli Studi di Torino Dipartimento di Scienze Mediche; Università degli Studi di Torino Dipartimento di Neuroscienze ‘Rita Levi Montalcini’: Universita degli Studi di Torino Dipartimento di Neuroscienze Rita Levi Montalcini; Universita degli Studi di Torino Dipartimento di Neuroscienze Rita Levi Montalcini; Università degli Studi di Torino Dipartimento di Scienze Mediche: Universita degli Studi di Torino Dipartimento di Scienze Mediche; Università degli Studi di Torino Dipartimento di Scienze Mediche: Universita degli Studi di Torino Dipartimento di Scienze Mediche; Università degli Studi di Torino Dipartimento di Scienze Mediche: Universita degli Studi di Torino Dipartimento di Scienze Mediche; Università degli Studi di Torino Dipartimento di Scienze Mediche: Universita degli Studi di Torino Dipartimento di Scienze Mediche; Università degli Studi di Torino Dipartimento di Scienze Mediche: Universita degli Studi di Torino Dipartimento di Scienze Mediche; Università degli Studi di Torino Dipartimento di Scienze Mediche: Universita degli Studi di Torino Dipartimento di Scienze Mediche; University of Turin Department of Neurosciences Rita Levi Montalcini: Universita degli Studi di Torino Dipartimento di Neuroscienze Rita Levi Montalcini; Università degli Studi di Torino Dipartimento di Scienze Mediche: Universita degli Studi di Torino Dipartimento di Scienze Mediche; University of Turin Department of Neurosciences Rita Levi Montalcini: Universita degli Studi di Torino Dipartimento di Neuroscienze Rita Levi Montalcini

**Keywords:** Parkinson’s disease, orthostatic hypotension, disability milestones, adverse outcomes, ambulatory blood pressure monitoring, hypotensive episodes

## Abstract

**Purpose.:**

Neurogenic orthostatic hypotension (nOH) is a frequent non-motor feature of Parkinson’s disease (PD), associated with adverse outcomes. Recently, 24-hour ambulatory BP monitoring (ABPM) has been shown to diagnose nOH with good accuracy (in the presence of at least 2 episodes of systolic BP drop ≥ 15 mmHg compared to the average 24-h). This study aims at evaluating the prognostic role of ABPM-hypotensive episodes in predicting PD disability milestones and mortality and comparing it to well-defined prognostic role of nOH.

**Methods.:**

PD patients who underwent ABPM from January 2012 to December 2014 were retrospectively enrolled and assessed for the development of falls, fractures, dementia, bed/wheelchair confinement, hospitalization, mortality, during an up-to-10-year follow-up.

**Results.:**

Ninety-nine patients (male 74%; age: 64.0 ± 10.1 years; PD duration: 6.4 ± 4.0 years) were enrolled. At baseline, 38.4% of patients had ABPM-hypotensive episodes and 46.5% had bedside nOH.

At Kaplan-Meier analysis patients with ABPM-hypotensive episodes had an earlier onset of falls (p = 0.001), fractures (p = 0.004), hospitalizations (p = 0.009), bed/wheelchair confinement (p = 0.032), dementia (p = 0.001), and showed a shorter survival (8.0vs9.5 years; p = 0.009). At Cox regression analysis (adjusted for age, disease duration, Charlson Comorbidity Index, and H&Y stage at baseline) a significant association was confirmed between ABPM-hypotensive episodes and falls (OR:3.626; p = 0.001), hospitalizations (OR:2.016; p = 0.038), and dementia (OR:2.926; p = 0.008), while bedside nOH was only associated with falls (OR 2.022; p = 0.039) and dementia (OR:1.908; p = 0.048).

**Conclusion.:**

The presence of at least two ABPM-hypotensive episodes independently predicted the development of falls, dementia, and hospitalization, showing a stronger prognostic value than the simple bedside assessment.

## INTRODUCTION

Orthostatic hypotension (OH) may affect at least 30% of patients with Parkinson’s disease (PD) [1][2][3], potentially resulting in dramatic complications, such as falls, fractures, head trauma, wheelchair/bed confinement and hospitalization, with a significantly increased mortality risk [4][5]. Moreover, recent evidence underlines the possible association between OH and cognitive impairment and cerebral atrophy [6][7][8][9]. Current diagnostic criteria for OH rely on office blood pressure (BP) measurements demonstrating a sustained drop of at least 20 mmHg (systolic) or 10 mmHg (diastolic) after 3 min of standing from a supine position [10]. Although useful in clinical practice, this definition remains anchored to an artificial assessment that only partially recapitulates the complexity of circadian BP fluctuations occurring during daily living activities [11]. In fact, these criteria are affected by a limited reproducibility (79% in neurogenic OH (nOH) and 67% in non-neurogenic OH) [12][13], with a maximal level of error for BP measurements carried out during the afternoon [14]. Therefore, the presence of OH may be largely underestimated.

In this context, the 24-hour ambulatory blood pressure monitoring (ABPM), a validated technique for the assessment of arterial blood pressure [15], could represent a reliable instrument for the detection of OH and autonomic neuropathy (AN) [16][17]. A recent study from our group [18], based on the evaluation of several ABPM parameters, demonstrated that having more than 2 episodes of systolic drop ≥ 15 mmHg during the waking period, compared to the average 24-hours systolic BP, resulted in a reproducible measure for the diagnosis of nOH, yielding high accuracy and specificity values.

In this study we sought to investigate, in a large cohort of parkinsonian patients, the potential prognostic role of ABPM-hypotensive episodes in predicting mortality and specific OH-associated complications (falls, fractures, bed/wheelchair confinement, cognitive impairment), thus informing on possible different trajectories of PD progression. Moreover, we aim to evaluate their role in informing on key healthcare economic indicators such as the presence of hospitalizations. Finally, we compare them to office nOH, an established predictor of adverse outcomes [4][19].

## METHODS

We retrospectively evaluated a cohort of PD patients. In order to reach a long-term observational time, we included patients who underwent ABPM at our center during the period comprised between January 2012 and December 2014 and followed-up with periodic neurological assessment for at least three years.

### Cohort selection

Parkinsonian patients were screened for the following inclusion/exclusion criteria:

#### Inclusion criteria

Diagnosis of PD per the UK Brain Bank criteria [20]; stable dosage of dopaminergic and vasoactive (antihypotensive and/or anti- hypertensive) medications for at least 4 weeks prior to ABPM; presence of ABPM and OH assessment, performed between January 2012 and December 2014.

#### Exclusion criteria

Chronic heart failure, chronic renal failure, diabetes mellitus, amyloidosis, autoimmune disorders, malignancies, or other diseases potentially associated with secondary forms of autonomic dysfunction [21]. Patients without constant periodic neurological follow-up (at least every 12–18 months), and/or complete medical records, and/or follow-up duration of at least 3 years (except for patients dead before the 3-year threshold) were also excluded.

### Baseline Evaluation

#### Clinical and neurological evaluation

All patients underwent a standardized clinical evaluation including medical history collection, vital parameters, complete physical examination, and PD staging by means of the Hoehn and Yahr (H&Y) scale [22]. Finally, Charlson comorbidity index was assessed [23].

#### Office blood pressure measurement

Office BP evaluations were carried out between 2 PM and 5 PM, at least 2 h after a meal, in a standardized environment at a room temperature of 71–75° Fahrenheit. BP values were collected in the supine position (average of the last three BP stable measurements) and every minute during the active standing position with an OMRON automatic sphygmomanometer (HEM-9219T-E, Japan ©). Heart rate (HR) values were also collected. OH was defined as a sustained systolic BP drop ≥ 20 mm Hg or a diastolic BP drop ≥ 10 mm Hg within 3 min of orthostatic test [10]. We included in the OH analyses only patients with nOH, defined as having a Δ HR/Δ systolic BP ratio < 0.5 beats per minute (bpm)/mmHg [24].

#### Ambulatory Blood Pressure Monitoring

Twenty-four hours ABPMs were performed with a portable device (Spacelabs 90207, Spacelabs Inc., Redmond, WA, USA ©) with appropriate cuff size placed on the non-dominant arm as per the current guidelines [15]. BP measurements were taken every 15 min during the daytime and nighttime. Patients were asked to keep a diary of occupational activities, sleep, and awake time, as well as the time of meals.

We used normal reference thresholds for ABPM and adhered to the definition of weighted BP variability and dipping patterns proposed by the European Society of Hypertension [15]. According to a previous study from our group [18] we defined hypotensive episode (Hypo-ep^Δ15/24h^) a systolic drop ≥ 15 mmHg compared to the average 24-h systolic BP, during the waking period (from awakening to lunch). We considered as significant the presence of at least two hypotensive episodes [18].

#### Follow-up evaluation

We retrospectively reviewed the medical records of each participant. For every outpatient visit we evaluated: the medical history, which included information on treatment, recent falls, fractures, ER accesses and hospitalizations; the neurological evaluation, including the staging of PD motor severity assessed by means of the H&Y scale; and the cognitive status. Dementia was diagnosed when Montreal Cognitive Assessment (MoCA) score was lower than 21/30 [25], or when reported at recent comprehensive neuropsychological assessment, or in the presence of incontrovertible symptoms/signs of cognitive impairment associated with functional limitation (e.g., executive or amnestic impairment needing assistance in common activities of daily living) [26]. Mortality data were searched in our electronic archives, which are constantly updated by the regional registry office.

#### Outcome Measures and Statistical analysis

The potential prognostic role of ABPM hypotensive episodes (Hypo-ep^Δ15/24h^) in predicting specific complications, was compared with a well-established predictor, such as office nOH.

Primary endpoints included mortality for all causes, falls, fractures, and cognitive impairment. Secondary endpoints included hospitalizations, and bed or wheelchair confinement.

Cumulative survival for each adverse outcome was evaluated through Kaplan-Meier curves. The sample was divided in patients with and without the following predictors: a) at least two hypotensive episodes (Hypo-ep^Δ15/24h^) at the ABPM during the waking period; b) office nOH. Differences between the two groups were assessed by means of the log-rank test. The prognostic role of the same predictors was then evaluated through Cox regression analysis, adjusting for age, Charlson comorbidity index, PD duration and severity (H&Y scale). In both Kaplan-Meier and Cox regression analyses, patients already reporting the adverse outcome at baseline (e.g., patients reporting falls or dementia) were excluded.

Continuous variables were expressed as mean ± standard deviation. Qualitative variables were expressed as frequencies or percentage values. Normal distribution of continuous variables was tested using the Shapiro-Wilk test. Differences between two independent groups were evaluated using a Student’s t-test for continuous variables with normal distribution and the Mann-Whitney test for continuous variables with non-normal distribution. Categorical variables were compared using the chi-square test or Fisher’s exact test according to sampling number of analysed groups. Statistical analysis was performed with SPSS (IBM Corp. Released 2017. IBM SPSS, Version 25.0. Armonk, NY: IBM Corp). Statistical significance was considered for p values < 0.05.

The present study was approved by the Institutional Review Committee of Turin (Comitato Etico Interaziendale A.O.U. Città della Salute e della Scienza di Torino – A.O. Ordine Mauriziano). All subjects submitted their written informed consent.

## RESULTS

Inclusion criteria were fulfilled by 143 patients; 44 patients presented exclusion criteria (n = 3 had chronic heart failure, n = 4 had chronic renal failure, n = 12 had diabetes mellitus, n = 12 had incomplete clinical data at baseline, n = 13 had incomplete clinical data at follow-up, or were lost at follow-up). Therefore, the study population consisted of 99 patients, with a higher prevalence of men (74%), a mean age of 64.0 ± 10.1 (range 34–79) years, and PD duration of 6.4 ± 4.0 (range 1–18) years. The average follow-up duration was 5.9 ± 2.7 years (range 1–10).

At baseline, 45 patients (45.5%) presented with H&Y stage 1, 38 (38.4%) with stage 2, 15 (15.2%) with stage 3, and one (1.0%) with stage 4, while no patients met the clinical criteria of H&Y stage 5. Previous history of arterial hypertension was present in the 29.3% of patients (n = 29). All patients were on dopaminergic treatment, with a levodopa equivalent daily dose (LEDD) of 662.0 ± 352.7 mg, while the 31.3% (n = 31) of the cohort was on vasoactive drugs (antihypertensive (n = 31) and/or antihypotensive (n = 24) medications).

OH was detected in 48 patients, but in two cases the Δ HR/ Δ BP ratio indicated the presence of non-neurogenic OH. The remaining 46 nOH patients (46.5%) were included in the analyses.

ABPM detected at least two hypotensive episodes (≥ 2 Hypo-ep^Δ15/24h^) in 38 patients (38.4%). Clinical and demographical characteristics of patients with and without hypotensive episodes at ABPM are shown in Table 1. Sensibility (67.4%), specificity (86.8%), and accuracy (77.8%) of the Hypo-ep^Δ15/24h^ criterion for the detection of nOH were comparable to those found in our previous study on a different PD population [18], confirming the consistency of the method.

Patients with hypotensive episodes were older and treated with higher doses of dopaminergic therapy; they had 3-fold higher prevalence of nOH (p < 0.001) and 2-fold higher prevalence of arterial hypertension (p = 0.027). Moreover, they showed higher nocturnal BP values (p = 0.001), BP loads (p = 0.001) and BP variability (p < 0.001), together with a 3-fold higher prevalence of reverse dipping pattern (p = 0.001) (Table 1).

At baseline, the prevalence of falls in the entire sample was 14.1% (n = 14), significantly higher in patients with hypotensive episodes at ABPM (26.3% vs. 6.6%, p = 0.008). Dementia was reported in 10.1% of patients (n = 10), with a 2-fold higher prevalence (15.8% vs. 6.6%) among patients with hypotensive episodes at ABPM, not reaching the statistical threshold (p = 0.128). No patient was bedridden/wheelchair-bound or reported recent fractures or hospitalization.

### Outcome evaluation

During the follow-up, we observed a high incidence of falls (40.0%; 34/85, excluding patients already reporting falls at baseline), dementia (37.1%; 33/89, excluding patients already demented at baseline), fractures (22.2%; 22/99), hospitalization (32.3%; 32/99), and bed or wheelchair confinement (13.1%; 13/99). Mortality rate was 13.1% (13/99), with a mean survival time from baseline of 5.7 ± 2.7 years and a mean disease duration at death of 15.3 ± 6.1 years. Both incidence and prevalence of falls were significantly higher in patients with ABPM hypotensive episodes (Incidence: 57.1% vs. 31.6%, p = 0.022; Prevalence: 68.4% vs. 36.1%, p = 0.002). The same observations were found for dementia (Incidence: 53.1% vs. 28.1%, p = 0.017; Prevalence: 60.5% vs. 32.8%, p = 0.006). Patients with ABPM hypotensive episodes presented with significant higher incidence of fractures (34.2% vs. 14.8%, p = 0.023), while for hospitalization and bed/wheelchair confinement the difference did not reach full statistical significance (42.1% vs. 26.2%, p = 0.078; and 21.1% vs. 8.2%, p = 0.064; respectively). Mortality was higher in patients with ABPM hypotensive episodes, with a trend towards statistical significance (21.1% vs. 8.2%, p = 0.064).

**Figure 1:**
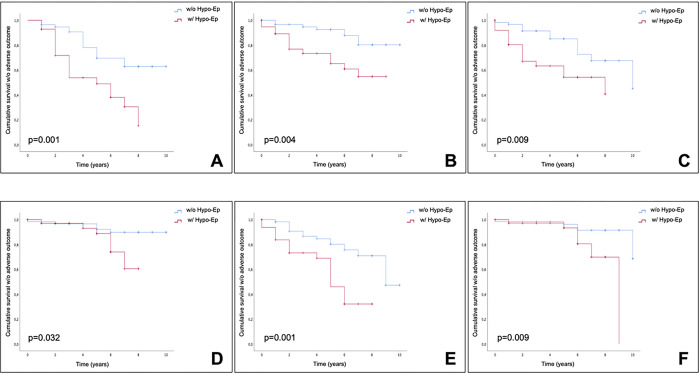
Hypotensive episodes at Ambulatory Blood Pressure Monitoring were associated with earlier onset of falls (A), fractures (B), hospitalizations (C), bedridden/wheelchair confinement (D), dementia (E), and shorter survival (F) (see manuscript for details). w/o: without; w/: with; Hypo-ep: at least 2 hypotensive episodes at Ambulatory Blood Pressure Monitoring

The Kaplan-Meier analysis revealed that the presence of hypotensive episodes at ABPM was associated with earlier onset of falls (4.9 years (95% CI 3.8–6.0) vs. 7.9 years (7.1–8.7); p = 0.001), fractures (6.4 years (95% CI 5.3–7.6) vs. 9.0 years (8.4–9.6); p = 0.004), hospitalizations (5.3 years (95% CI 4.1–6.4) vs. 8.2 years (95% CI 7.4–9.0); p = 0.009), bedridden/wheelchair confinement (7.1 years (95% CI 6.6–7.7) vs. 9.4 years (95% CI 8.8–9.9); p = 0.032), and dementia (5.0 years (95% CI 4.0–6.0) vs. 8.1 years (7.2–8.9); p = 0.001). Patients with hypotensive episodes at ABPM also showed a shorter survival (8.0 years (95% CI 7.3–8.7) vs. 9.5 years (95% CI 9.0–10.0); p = 0.009) (Fig. 1).

**Figure 2:**
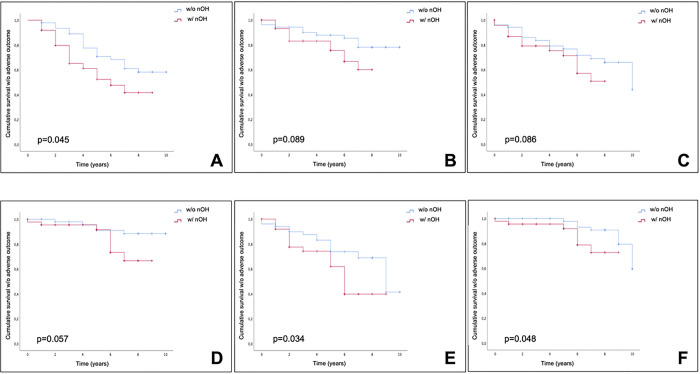
nOH detected at office evaluation was associated with earlier onset of falls (A), dementia (E), and shorter survival (F), while no association was present for fractures (B), hospitalizations (C), and bedridden/wheelchair confinement (D) (see manuscript for details). w/o: without; w/: with; nOH: neurogenic orthostatic hypotension

Office OH was related to earlier onset of falls (5.8 years (95% CI 4.7–7.0) vs. 7.7 years (6.9–8.6); p = 0.045), earlier development of cognitive impairment (6.0 years (95% CI 5.0–7.0) vs. 7.8 years (95% CI 6.9–8.7); p = 0.034), and death (8.0 years (95% CI 7.3–8.7) vs. 9.5 years (9.1–10.0); p = 0.048), while a trend towards statistical significance was observed for hospitalizations (p = 0.086), fractures (p = 0.089), and bedridden/wheelchair confinement (p = 0.057) (Fig. 2).

After correction for potential confounders, such as age, Charlson comorbidity index, PD duration, and PD severity at baseline, the Cox-Regression analysis showed a significant association between the presence of hypotensive episodes at ABPM and falls (OR 3.626, 95% CI 1.7–7.6; p = 0.001), hospitalizations (OR 2.016, 95% CI 1.2–4.5; p = 0.038), and dementia (OR 2.926, 95% CI 1.3–6.5; p = 0.008); the association with fractures (OR 2.155, 95% CI 0.9–5.4; p = 0.092) showed a trend towards statistical significance, while no association was found with bed/wheelchair confinement (OR 2.462, 95% CI 0.7–8.5; p = 0.155) or mortality (OR 1.352, 95% CI 0.4–5.1; p = 0.655).

**Figure 3:**
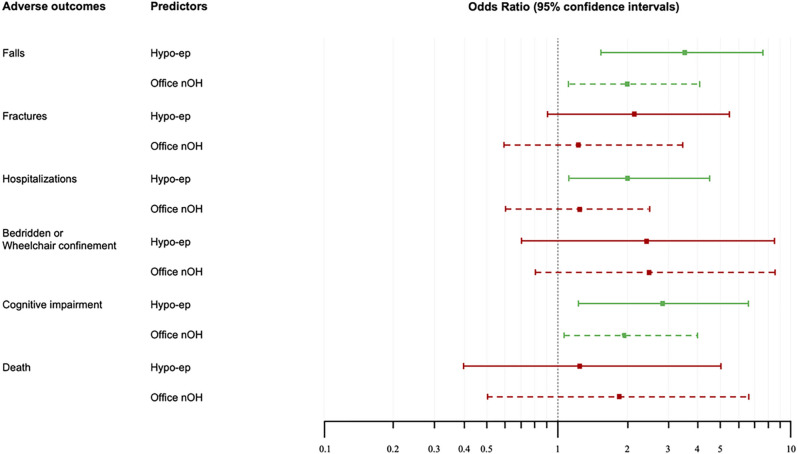
Hypotensive episodes at Ambulatory Blood Pressure Monitoring, even after correcting for potential confounders, were significantly associated with falls, hospitalization, and dementia; nOH detected at the office evaluation was associated only, and with lower power and significance, with falls and dementia (see manuscript for details). Hypo-ep: hypotensive episodes at Ambulatory Blood Pressure Monitoring; nOH: neurogenic orthostatic hypotension; Continuous line: Hypotensive episodes confidence intervals; dotted line: neurogenic orthostatic hypotension confidence intervals; green line: significant association between predictor and adverse outcome; red line: non-significant association between predictor and adverse outcome

After the same corrections, office nOH showed inferior prognostic value for the adverse outcomes considered, only presenting a significant association with falls (OR 2.022, 95% CI 1.2–4.1; p = 0.039) and dementia (OR 1.908, 95% CI 1.1–4.0; p = 0.048). Details are presented in Fig. 3.

## DISCUSSION

OH is a frequent and detrimental feature of PD; however, in clinical practice its diagnosis is still overlooked. One of the main features limiting a correct OH detection in an outpatient setting is the presence of BP circadian fluctuations. In this context, we explored the prognostic role of hypotensive episodes detected at the ABPM in predicting mortality and specific adverse outcomes in a large cohort of parkinsonian patients.

We retrospectively evaluated mortality and the occurrence of important milestones of disability during an up-to-10-year follow-up and compared them between patients with and without ABPM hypotensive episodes. Finally, we evaluated the same differences between patients with and without office nOH.

We found a significant association between ABPM hypotensive episodes and earlier onset of falls, cognitive impairment, hospitalizations, fractures, being bedridden or wheelchair-bound, and mortality. After correcting for potential confounders, hypotensive episodes retained a significant correlation with higher risk of falls, hospitalizations, and cognitive impairment. On the other hand, office nOH was associated with earlier onset of falls, cognitive impairment, and death, but only falls and cognitive impairment maintained the association after the adjusted analysis.

Association with falls. nOH is a well-known risk factor for falls in general population, and it is considered one of the main issues to be targeted to reduce the risk of falls, together with functional status, home safety, and vision disturbances [27]. In PD patients, OH is independently associated with an up-to-10-fold higher risk ok falls [5]. However, daily BP profile is characterized by significant circadian fluctuations, and is influenced by many intrinsic and extrinsic features, such as sleep, temperature, meals, fluid intake, exercise, evaluation setting (e.g., in-hospital vs. home), and drugs intake. Therefore, the simple in-office evaluation of OH could be burdened by a certain amount of inaccuracy, while the evaluation by means of ABPM could led to a more detailed identification of hypotensive episodes, besides providing important information on supine hypertension, a frequently overlooked feature of cardiovascular dysautonomia [28]. Our results seem to confirm this assumption, since both ABPM episodes and office OH were associated with higher risk of falls, but the former showed a stronger and more significant correlation.

Association with cognitive impairment. The association of OH and cognitive impairment has been detected both in general population and in patients with Alzheimer’s Disease or vascular dementia [29] [30][31]. Large population studies highlighted an increased risk of developing dementia in patients with OH, ranging from 1.2-to-2-fold [32]. This association results even stronger in α-synucleinopathies, with a 3-to-8-fold higher risk of cognitive impairment in PD patients with OH [6][32][33].

Possible pathophysiological mechanisms are multiple and still to be fully clarified [8]. While some authors postulate a phenotypical association between dysautonomia, cognitive impairment, and worse motor impairment [34], other works reported that repeated bouts of cerebral hypoperfusion, along with altered regional patterns in supine cerebral blood flow and the presence of supine hypertension, could lead to greater burden of white matter lesions and cerebral atrophy, thus favoring the development of cognitive impairment [35][9][32][36]. However, these two hypotheses are not mutually exclusive, and both diffuse pathology and cerebral hypoperfusion may play a synergistic role [9]: the possibility exists that, in the context of a diffuse neurodegeneration, chronic OH and impaired dynamic cerebral auto-regulation [37] contribute to the worsening of neuropathology, probably by means of hypoxia-induced neurodegeneration, rather than by favoring α-synuclein deposition or vascular pathology [38]. In our study, we observed a significant association between ABPM hypotensive episodes (and in-office OH) and dementia, even after adjusting our analyses for many confounding variables that may explain the phenotypical association between OH and dementia (i.e., age, Charlson comorbidity index, PD duration and motor severity), thus suggesting a prevalent causative or synergistic role of OH in determining cognitive impairment. This observation seems in line with the recent findings by Ruiz Barrio and colleagues [38], who in a large pathological study on PD and Multiple System Atrophy patients observed an independent association between OH and cognitive impairment, but did not find differences in α-synuclein, β-amyloid, or tau pathology in patients with and without OH. Finally, in the present study, the strength and the significance of the association with cognitive impairment was greater for ABPM hypotensive episodes than office OH, further confirming the usefulness of ABPM in capturing the complexity of the circadian fluctuation of BP, and suggesting a possible role of BP variability in determining worse functional outcomes [39].

Association with hospitalization. About 80,000 hospitalizations in the United States each year are related to OH, mainly in the elderly population [40][41]. In PD patients, OH is an independent determinant of greater health care utilization [1], both for the potential consequences of the hypotensive episodes (falls, syncopes, head injury, fractures) and for the presence of other clinical features potentially responsible for hospitalization, which usually coexist with OH, such as ageing, diabetes, and cardiovascular comorbidities [42]. In our sample, ABPM hypotensive episodes were significantly associated with a 3-year earlier need for hospitalization and a 2-fold higher risk of hospitalization, compared to patients without episodes. Surprisingly, office OH did not show significant correlation at the adjusted Cox regression analysis, possibly due to the long-term follow-up in a relatively aged population, which resulted in a high probability of observing hospitalization for causes other than OH.

Association with fractures and bed/wheelchair confinement. Although univariate Kaplan-Meier analysis showed earlier occurrence of fractures and bed or wheelchair confinement in patients with hypotensive episodes or office OH, similarly to previous reports [43][44], these observations were not confirmed at the corrected analysis, probably due to the inclusion, among the considered confounders, of age, disease duration, and motor disability, which are well-known features associated with these detrimental disability milestones in PD and other α-synucleinopathies [45][46][47].

Association with mortality. Autonomic dysfunction [48] and, specifically, OH [49] have been associated with shorter survival in PD. In our sample, mortality was significantly associated with ABPM episodes and OH, with a 1.5-year shorter survival. However, the corrected analyses did not confirm this association, probably due to the relatively low rate of death during the follow-up.

The main strengths of our study include the long-term follow-up, with seriate and standardized clinical evaluations, as well as rigorous patients’ selection. However, some limitations should be taken into account. First, the relatively low sample size. Second, the retrospective design may have hampered the collection of important clinical data, including the disability milestones considered in the study, although we included only patients with a periodic and standardized follow-up in order to minimize this possible bias. Third, the absence of seriate collection of office OH or ABPM data, may have underestimated the incidence of new OH; however, if this were the case, we would have probably observed an even greater association between hypotensive episodes and disability milestones or mortality. Fourth, the definition of nOH was not based on cardiovascular autonomic test assessment, although the application of the recently proposed Δ HR/Δ systolic BP index [24] should have attenuated this bias. Fifth, the lack of a comprehensive evaluation of the autonomic symptoms other than OH limits firm considerations on a causal relationship between hypotensive episodes/OH and the development of disability milestones.

In conclusion, our findings highlight the potential role of repeated hypotensive episodes in determining worse outcomes in PD patients, confirming and strengthening, by means of a 10-year follow-up, previous Literature data. Though, our study introduces a novel element that has so far received little consideration. In fact, we demonstrated the usefulness of ABPM in predicting detrimental PD complications, with apparent greater accuracy compared to simple OH bedside evaluation, due to its higher capability in capturing the complexity of BP circadian fluctuations. Thus, a wider application of ABPM, both in clinical practice and in research context, is warranted to enhance the comprehension of the relationship between cardiovascular dysautonomia and PD progression trajectories.

## Figures and Tables

**Table 1:** Demographic and clinical characteristics.

	Overall *(n. 99)*	w/ Hypo-ep *(n. 38)*	w/o Hypo-ep *(n.61)*	*p-value*
Age *[years]*	64.0 ± 10.1	67.8 ± 10.1	61.7 ± 9.5	**0.003**
Female sex *[n.] (%)*	26 (26.3)	10 (26.3)	16 (26.2)	*0.992*
Disease duration *[years]*	6.4 ± 4.0	7.1 ± 4.4	5.9 ± 3.6	*0.163*
Follow-up duration *[years]*	5.9 ± 2.7	5.2 ± 2.5	6.3 ± 2.7	**0.035**
Total LEDD *[mg]*	662.0 ± 352.7	757.1 ± 381.7	602.8 ± 322.6	**0.034**
Vasoactive therapy *[n.] (%)*	31 (31.3)	17 (44.7)	14 (22.9)	**0.023**
Previous arterial hypertension *[n.] (%)*	29 (29.3)	16 (42.1)	13 (21.3)	**0.027**
nOH *[n.] (%)*	46 (46.5)	31 (81.6)	15 (24.5)	**< 0.001**
**Ambulatory blood pressure monitoring**				
24h SBP *[mmHg]*	119.7 ± 10.4	121.5 ± 9.9	118.6 ± 10.6	*0.191*
24h MBP *[mmHg]*	88.1 ± 7.8	89.5 ± 7.6	87.3 ± 7.8	*0.170*
24h DBP *[mmHg]*	71.8 ± 7.5	72.3 ± 7.6	71.4 ± 7.4	*0.541*
Daytime SBP *[mmHg]*	121.6 ± 10.9	121.5 ± 10.0	121.7 ± 11.5	*0.949*
Daytime MBP *[mmHg]*	89.9 ± 8.6	90.0 ± 8.1	90.0 ± 8.9	*0.968*
Daytime DBP *[mmHg]*	73.9 ± 8.0	73.4 ± 7.9	74.2 ± 8.1	*0.664*
Daytime HR *[bpm]*	76.8 ± 9.5	76.8 ± 11.0	76.8 ± 8.5	*0.982*
Nighttime SBP *[mmHg]*	115.2 ± 14.7	122.1 ± 16.5	110.9 ± 11.7	**0.001**
Nighttime MBP *[mmHg]*	83.8 ± 11.0	89.1 ± 12.4	80.5 ± 8.5	**< 0.001**
Nighttime DBP *[mmHg]*	66.9 ± 9.8	70.7 ± 11.5	64.6 ± 7.7	**0.006**
Nighttime HR *[bpm]*	65.3 ± 8.9	67.3 ± 9.3	64.1 ± 8.6	*0.087*
Daytime SBP load *[%]*	19.7 ± 20.9	21.4 ± 18.2	18.7 ± 22.6	*0.528*
Daytime DBP load *[%]*	17.0 ± 19.0	18.1 ± 17.6	16.4 ± 19.9	*0.653*
Nighttime SBP load *[%]*	33.7 ± 33.5	49.2 ± 36.7	24.0 ± 27.4	**0.001**
Nighttime DBP load *[%]*	34.0 ± 31.3	47.6 ± 35.1	25.5 ± 25.4	**0.001**
Reverse dipping *[n.] (%)*	30 (30.3)	19 (50.0)	11 (18.0)	**0.001**
w-BPV *[mmHg]*	12.1 ± 3.8	14.6 ± 4.5	10.6 ± 2.2	**< 0.001**
Hypo-ep^Δ15/24 h^ *[n.]*	2.1 ± 3.3	5.2 ± 3.5	0.3 ± 0.4	**< 0.001**
**Office blood pressure values**				
SBP (supine) *[mmHg]*	130.6 ± 16.5	140.0 ± 17.1	124.8 ± 13.3	**< 0.001**
DBP (supine) *[mmHg]*	79.1 ± 8.9	82.9 ± 8.5	76.7 ± 8.3	**< 0.001**
HR (supine) *[bpm]*	75.8 ± 11.3	77.8 ± 12.8	74.5 ± 10.2	*0.151*
SBP (orthostatism 1’) *[mmHg]*	116.0 ± 21.0	118.2 ± 25.2	114.6 ± 17.9	*0.405*
DBP (orthostatism 1’) *[mmHg]*	74.9 ± 11.6	76.2 ± 12.8	74.1 ± 10.9	*0.389*
SBP (orthostatism 3’) *[mmHg]*	115.5 ± 18.4	116.8 ± 21.8	114.8 ± 16.0	*0.600*
DBP (orthostatism 3’) *[mmHg]*	74.7 ± 10.7	75.0 ± 12.1	74.5 ± 9.9	*0.825*
HR (orthostatism) *[bpm]*	84.8 ± 13.2	85.2 ± 15.3	84.5 ± 11.8	*0.817*

LEDD: levodopa equivalent daily dose; nOH: neurogenic orthostatic hypotension; SBP: systolic blood pressure; MBP; mean blood pressure; DBP: diastolic blood pressure; BPV: blood pressure variability; Hypo-ep^Δ15/24 h^: hypotensive episode at Ambulatory Blood pressure Monitoring; HR: heart rate; w-BPV: weighted blood pressure variability; w/: with; w/o: without
